# North American and International Students’ Perspectives on Older Adults

**DOI:** 10.1093/geroni/igaa057.1044

**Published:** 2020-12-16

**Authors:** Adam Shea, Jessica Strong, Kirsten Graham

**Affiliations:** 1 university of prince edward island, Charlottetown, Prince Edward Island, Canada; 2 University of Prince Edward Island, Charlottetown, Prince Edward Island, Canada; 3 Southern Utah University, Cedar City, Utah, United States

## Abstract

Ageism is recognized around the world as detrimental to older adults’ health and well-being, and there are differences in how cultures view older adults. Infrequently are ageist attitudes among cultures compared within one study. Here, we sought to examine views on older adults across cultures in a sample of university students attending school in North America (n=31). As part of a larger survey of ageist attitudes, we conducted a thematic analysis on open-ended responses to the question “How are older adults viewed in your culture?” Half of the respondents were international students. Results found similarities and differences between groups. First, both groups saw older adults as individuals who are and should be respected. Second, however, North American students viewed older adults as “important” and “role models”, whereas international students viewed older adults as “leaders” of and at the “head of the family”. Third, North American students saw older adults as “needing extra help.” In contrast, international students reported that families “should provide care” as a duty or responsibility. Fourth, North American students believed older adults provide “wisdom,” “love,” and “support” but the international students felt older adults provided knowledge, experience, and opinions that are valued by the family and society that are important to decision making. Finally, North American students describe negative perceptions and experiences with older adults, which was completely absent from the international students responses. Results are discussed in a cultural context of personal and formal relationships with elders.

